# Evolutionary genomics can improve prediction of species’ responses to climate change

**DOI:** 10.1002/evl3.154

**Published:** 2020-01-14

**Authors:** Ann‐Marie Waldvogel, Barbara Feldmeyer, Gregor Rolshausen, Moises Exposito‐Alonso, Christian Rellstab, Robert Kofler, Thomas Mock, Karl Schmid, Imke Schmitt, Thomas Bataillon, Outi Savolainen, Alan Bergland, Thomas Flatt, Frederic Guillaume, Markus Pfenninger

**Affiliations:** ^1^ Senckenberg Biodiversity and Climate Research Centre Frankfurt am Main Germany; ^2^ Department of Plant Biology Carnegie Institution for Science Stanford California; ^3^ Swiss Federal Institute WSL Birmensdorf Switzerland; ^4^ Institute of Population Genetics Vetmeduni Vienna Austria; ^5^ School of Environmental Sciences University of East Anglia Norwich United Kingdom; ^6^ Institute of Plant Breeding, Seed Science and Population Genetics University of Hohenheim Stuttgart Germany; ^7^ Institute of Ecology, Evolution and Diversity Goethe‐University Frankfurt am Main Germany; ^8^ LOEWE Centre for Translational Biodiversity Genomics (LOEWE‐TBG) Frankfurt am Main Germany; ^9^ Bioinformatics Research Center Aarhus University Aarhus Denmark; ^10^ Department of Ecology and Genetics University of Oulu Finland; ^11^ Department of Biology University of Virginia Charlottesville Virginia; ^12^ Department of Biology University of Fribourg Fribourg Switzerland; ^13^ Department of Evolutionary Biology and Environmental Studies University of Zürich Zürich Switzerland; ^14^ Institute for Organismic and Molecular Evolution Johannes Gutenberg University Mainz Germany

**Keywords:** Biodiversity loss, eco‐evolutionary dynamics, genomic quantitative genetics, models

## Abstract

Global climate change (GCC) increasingly threatens biodiversity through the loss of species, and the transformation of entire ecosystems. Many species are challenged by the pace of GCC because they might not be able to respond fast enough to changing biotic and abiotic conditions. Species can respond either by shifting their range, or by persisting in their local habitat. If populations persist, they can tolerate climatic changes through phenotypic plasticity, or genetically adapt to changing conditions depending on their genetic variability and census population size to allow for de novo mutations. Otherwise, populations will experience demographic collapses and species may go extinct. Current approaches to predicting species responses to GCC begin to combine ecological and evolutionary information for species distribution modelling. Including an evolutionary dimension will substantially improve species distribution projections which have not accounted for key processes such as dispersal, adaptive genetic change, demography, or species interactions. However, eco‐evolutionary models require new data and methods for the estimation of a species' adaptive potential, which have so far only been available for a small number of model species. To represent global biodiversity, we need to devise large‐scale data collection strategies to define the ecology and evolutionary potential of a broad range of species, especially of keystone species of ecosystems. We also need standardized and replicable modelling approaches that integrate these new data to account for eco‐evolutionary processes when predicting the impact of GCC on species' survival. Here, we discuss different genomic approaches that can be used to investigate and predict species responses to GCC. This can serve as guidance for researchers looking for the appropriate experimental setup for their particular system. We furthermore highlight future directions for moving forward in the field and allocating available resources more effectively, to implement mitigation measures before species go extinct and ecosystems lose important functions.

**Impact Summary**

Global climate change (GCC) will lead to severe environmental changes and many species will lose their habitats. According to the recent Global Assessment Report on Biodiversity and Ecosystem Services (IPBES 2019), 5% of all species are at risk of extinction from 2°C global warming alone. To escape demographic decline or extinction, species have three different strategies to respond to changing environmental conditions: (1) range shift to track their ecological niche, (2) phenotypic plasticity to tolerate environmental change, and (3) genetic evolution to adapt to new local conditions. The ability to disperse depends on species specific characteristics, for example, birds can fly, whereas trees are sessile and, thus, have to cope with their local conditions. The ability of a species to genetically adapt to changing conditions depends on species‐specific characteristics such as genetic variability and population size. If we want to counteract species extinction by conservation strategies, we first need to be able to predict how different species will respond to GCC. A key element for accurate predictions is the potential for dispersal and/or evolutionary response of as many species as possible, and particularly of so‐called keystone species which are of major importance for their respective ecosystem.

Here, we argue that it is possible to use genomic data to understand a species’ evolutionary potential and then use this to improve prediction models that can reliably predict how species will respond to changing climate conditions across their distribution area. A lot has already been learned about the genomic footprints of adaptation to climate in different organisms and we highlight some important research strategies. Similarly, there have been great advances in prediction modelling. So‐called eco‐evolutionary models are most promising for successfully integrating ecological and evolutionary information. However, these models depend on large amounts of ecological and genomics (i.e., evolutionary) data. Scientists will therefore have to establish large consortia and engage with local communities to collect the required data before the consequences of global climate change are irreversible.

## A Global Evolutionary Challenge

**Figure 1:**
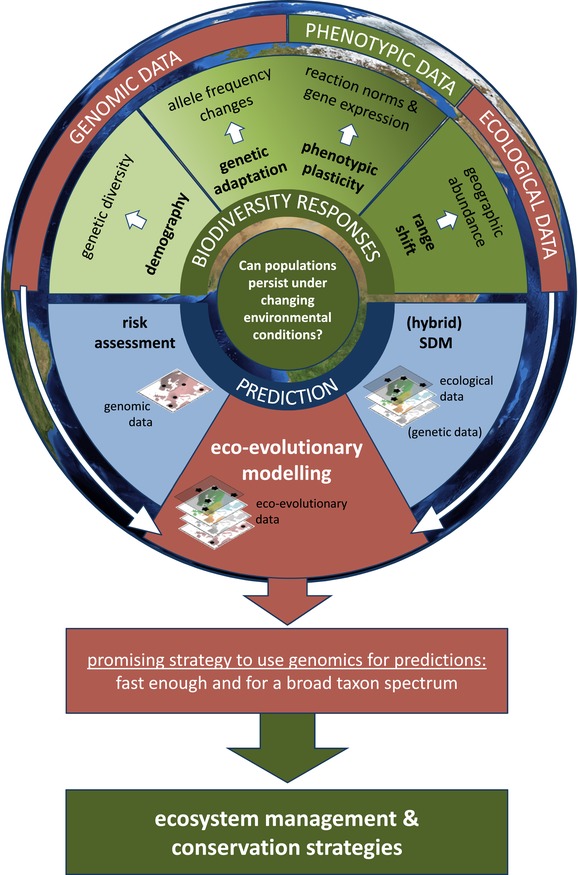
Graphical outline of how biodiversity can respond to changing environmental conditions under GCC, how to investigate these mechanisms, and how different types of empirical data can be used for predicting biodiversity responses to climate change (read from centre to top, then from centre to bottom). Sections in red highlight the connection of genomic and ecological data as basis for eco‐evolutionary modelling as most promising strategy to generate predictions for a relevant proportion of biodiversity fast enough to meet the accelerating pace of GCC. For a final implementation of this strategy, there is further demand for the development of tools to reliably estimate fitness from cohort/time‐series data. Predictions of how biodiversity responds to GCC are fundamental to urgently needed strategies for ecosystem management and conservation in order to counter‐act the imminent loss of biodiversity, ecosystem functioning, and ecosystem services.

Global climate change (GCC) is proceeding at an unprecedented rate and has major ecological consequences. Changes in temperature regimes or precipitation patterns strongly impact local environmental conditions of a species’ habitat (Walther et al. 2002; Root et al. 2003; Parmesan 2006). Consequently, the overall question is whether and how species can cope with the accelerated rate of environmental change. There are following four general and mutually nonexclusive strategies on how species and their populations may respond to changes of local environmental conditions (Fig. 1): (1) shifting their distribution range (niche tracking), (2) persisting in their local habitat because they are phenotypically plastic enough to tolerate environmental changes, (3) persisting in their local habitat by genetic adaptation to new conditions (niche evolution), and (4) persisting their ecological niche but experience demographic decline or even go extinct.

While GCC proceeds and ecological consequences are intensifying, conservation and management strategies to cope with changes in global biodiversity are lagging behind, because few studies investigate the response of species to GCC quantitatively and evolutionarily. We argue that genomic data can be used to infer the evolutionary potential for future adaptations of a relevant proportion of biodiversity and in due time. We outline research developments and advances in the field of GCC genomics, as well as in the field of predictive species distribution modelling. Furthermore, we encourage the establishment of international consortia, also involving society at large, to meet challenges in data collection and analysis. It is our objective to motivate future GCC research, to encourage the integration of GCC genomics with predictive modelling, and to generate comprehensive eco‐evolutionary data in the context of climate change to improve predictions of species’ potential to adapt to GCC.

## Investigating Biodiversity Responses to Climate Change

Identifying general features of biodiversity responses to climate change is a crucial but challenging mission (Fitzpatrick and Edelsparre 2018). Unravelling the underpinnings of evolutionary responses and, especially, of mechanisms of adaptation, can deliver the missing information, that is, “evolution,” for integrative models to improve the prediction of species responses to GCC.

### NICHE TRACKING

Due to the gradual variation of climate factors across geographic space, climatic changes can shift climatic conditions along gradients, excluding mountain tops, ocean currents, or terrestrial regions isolated by geographic barriers. If species are mobile enough or at least possess the ability to disperse their offspring across the borders of their local habitat (e.g., seed or larval dispersal), they have the potential to track their ecological niche by shifting their distribution range along the climate gradient. Evidence for such range shifts comes from studies investigating invasive species (Dukes and Mooney 1999; Fridley 2012; Wolkovich et al. 2013), shifts in breeding and overwintering ranges of migratory birds (Strode 2003; Rolshausen et al. 2009; Both et al. 2010; Zurell et al. 2018), as well as geographical range shifts in insects (Hill et al. 1999; Hickling et al. 2005; Crozier and Dwyer 2006) and plants (Walther et al. 2002; Thuiller et al. 2005; Kelly and Goulden 2008; Lenoir et al. 2008; Chen et al. 2011).

Generally, such investigations rely on multiple time points of occurrence/abundance data of populations across distribution ranges and might be extended by information of phenotypic differences among populations. However, catching, marking, and tracking a large number of individuals often involve logistical complications. Genetic tools provide a great variation of potential solutions to measuring dispersal, because dispersal leads to gene flow (reviewed in Broquet and Petit 2009).

### NICHE PERSISTENCE BY PHENOTYPIC PLASTICITY

As long as environmental changes do not exceed the physiological limits of an organism, individuals can persist by plastic responses. Plastic reprogramming of the unchanged genomic basis allows individuals to respond to environmental changes (Aubin‐Horth and Renn 2009). These responses can be investigated via acclimation and climate associated life‐history experiments with populations from different climate regimes. The aim is to obtain reaction norms, which give information on the degree of phenotypic plasticity versus genetic adaptation, and to be able to make predictions on the climate niche breadth of individual species as well as vulnerability in respect to GCC (Fangue et al. 2006; Calosi et al. 2008; Andrew et al. 2013; Foray et al. 2014; Gaitán‐Espitia et al. 2017). Investigations targeting plastic phenotypes have shed light on the molecular basis of cold and heat stress (Gleason and Burton 2015; Zhang et al. 2015; Xiao et al. 2016; Chou et al. 2018), desiccation resistance (Mizrahi et al. 2015; Menzel et al. 2018), and thermal tolerance (Mock et al. 2017; Gunderson et al. 2018; Hermann et al. 2018).

Since phenotypic plasticity, by definition, is the ability of one genotype to generate multiple phenotypes, genomic data need to be supplemented with experiments and other omics approaches such as transcriptomics, epigenomics, or proteomics (Aubin‐Horth and Renn 2009).

### NICHE EVOLUTION BY GENETIC ADAPTATION

Changes in environmental conditions are expressed as changes in the local selection regime acting on populations. Such altered selection regimes will eventually drive genetic adaptation. Evidence for genetic adaptation is manifold, including climate‐driven genetic changes in phenology and phenotype such as changes in the timing of vegetation development (Willis et al. 2008; Anderson et al. 2012; van Asch et al. 2013), reproduction (Bradshaw and Holzapfel 2001; Franks et al. 2007), seasonal range shifts (Pulido and Berthold 2003), body size (Daufresne et al. 2009; Gardner et al. 2011), and the strength of competition (Mboup et al. 2012; Bocedi et al. 2013).

With regard to long‐term physiological costs and fitness benefits, genetic adaptation is the most relevant scenario of species’ responses to climate change. Genomic data provides insight in the genomic basis underlying evolutionary adaptation and, thus, contribute most to understand mechanisms of genetic adaptation and to solve the problem of delivering clear evidence (Gienapp et al. 2008; Merilä 2012). To investigate the evolution of genetic adaptation under changing environmental conditions, knowledge of the initial genetic state, that is, the ancestral state, as well as the adapted/evolved state to the new conditions is necessary. To this end, the ancestral state is either approximated by correlation of environmental and genetic heterogeneity (space‐for‐time approach), or by directly following the evolutionary trajectory in time‐series data (time‐for‐time approach). Space‐for‐time approaches can inform on the amount of standing genetic variation present and on the genetic changes necessary to adapt from the current (initial) condition to a potential future condition. Time‐for‐time approaches inform on the likelihood of the change from the known initial condition.

#### Space‐for‐time approach

Studying the correlation between genomic and environmental variation across populations along climatic gradients, provides a first approximation of how much genetic change might be expected given future climate change projections (Rellstab et al. 2015; Li et al. 2017). *Environmental association analyses* (*EAAs*) provide a correlative insight in genotype‐environment interactions (Coop et al. 2010). EAAs are especially powerful when combined with traditional population genomic approaches to detect highly differentiated outlier loci.

Most genomic studies of climate adaptation to date are based on *reduced‐representation sequencing* of natural populations, that is, sequencing parts of an organism's DNA by RAD‐Seq, RNA‐Seq, targeted sequencing (e.g., exome capture), SNP‐chips, or the sequencing of previously known candidate genes (Hoban et al. 2016). The resulting genetic polymorphism data provide insight into the genetic variation of populations and can be used to identify signatures of selection and patterns of local adaptation (Savolainen et al. 2013). There is a growing list of studies that identify important candidate genes or loci involved in climate adaptation of nonmodel species (e.g., Jaramillo‐Correa et al. 2015; Pluess et al. 2016; Roschanski et al. 2016; Rellstab et al. 2017; Housset et al. 2018, Ahrens et al. 2019). Reduced‐representation sequencing is especially useful for landscape genomics studies with species having large genome sizes. For example in two distantly related conifer species (with genome sizes above 20 Gb), analysis using exome capture data of most of the coding region provided evidence of convergent adaptation to climate (Yeaman et al. 2016). However, studies relying on reduced‐representation sequencing approaches by definition only evaluate a small to moderate proportion of the genome and will, therefore, likely miss many signals of local adaptation (Lowry et al. 2017, but also see Catchen et al. 2017 or Benjelloun et al. 2019). There are additional limitations, such as some reduced‐representation approaches depend on previous knowledge of candidate loci, regions, or genes (e.g., SNP‐chips) and generally the link to fitness‐relevant phenotypic traits cannot be tested (Table 1).

**Table 1:** Experimental approaches to assess evolutionary responses to climate change using different sets of biological data. The column *data type/method* comprises different sequencing techniques and experimental setups. *Genomic resolution* outlines the detail of genomic information at which the genomic footprint of climate adaptation can be investigated in a *population genetics* or *quantitative genetics* context. *Inferable biological information* lists parameters that can be estimated applying the respective approach (cumulative across approaches with the complete parameter‐set inferable with the lower approach). Colour bars in the background visualise to what extend genomic information can be assessed for a broad range of taxa and contribute valuable information to be used in eco‐evolutionary prediction models. The two approaches with the best compromise of suitability are printed in bold

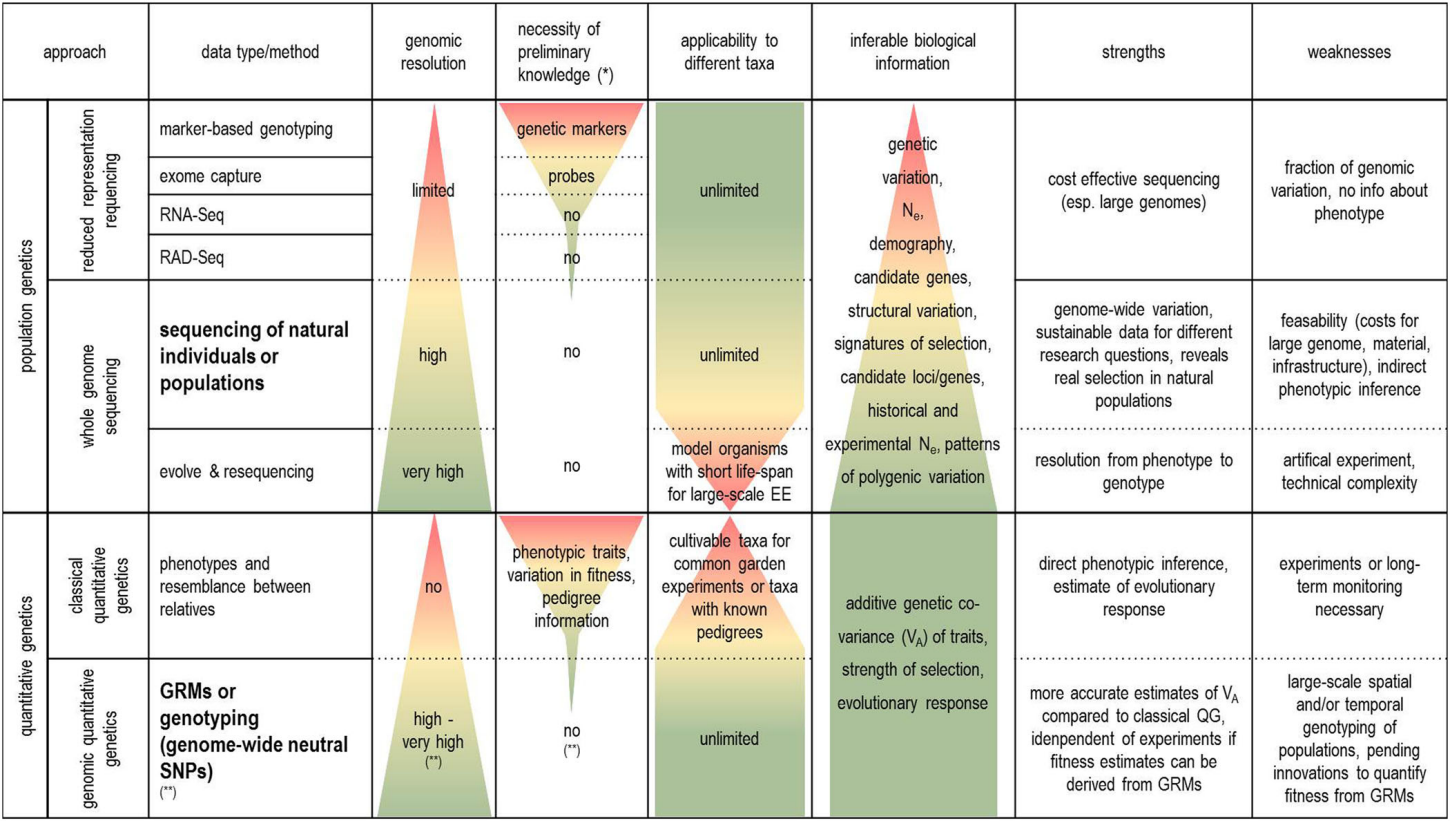	

(*) Does not include genomic resources, that is, reference genome with/without annotation.

(**) Ideally WGS data of cohorts or time‐series data that allow estimating relatedness as well as fitness from genomic relatedness matrices (GRMs), otherwise preliminary knowledge of phenotypic traits will be necessary and applicability will be restricted to taxa that are suitable for trait measuring.

In contrast, *whole genome sequencing* (WGS) delivers data on the entire genome of individuals or pooled individuals from a population (Pool‐Seq). When a reference genome is available, WGS provides information on the spatial pattern of variation along chromosomes. Genome‐wide information can be used to infer population history based on genome‐wide patterns of neutral genetic diversity, for the estimation of genome‐wide signatures of selection, or the analysis of variation in chromosome structure, or the genome‐wide landscape of differentiation and recombination (Table 1; Lexer and Stölting 2012; Hoban et al. 2016).

If EAAs are combined with WGS data, a comprehensive set of loci is used for correlation to environmental variables and thereby, depending on population structure and the strength of differentiation along the environmental gradient, minor effect loci can ideally also be uncovered (de Villemereuil et al. 2014). This approach requires WGS data of natural populations that should be distributed along environmental gradients. This is in principle applicable to all organisms with a small to medium genome size, a reference genome, even of suboptimal quality, and populations spanning a sufficiently steep environmental gradient. EAA do not require prior knowledge of specific phenotypic traits; they are, therefore, less labour intensive and will become affordable even for intermediate size genomes due to decreasing costs of sequencing technologies.

Conceptually, *genome‐wide association studies* (GWAS) are similar to EAA with the major difference that GWAS require both genomic information obtained from individuals and phenotypic measurements on certain traits (continuous or categorial; Balding 2006) on the same set of individuals. Because GWAS identifies associations between genomic regions and phenotypic traits, it is a powerful approach to uncover the genomic underpinnings of quantitative traits (such as phenotypic responses to most climate factors).

Investigations applying EEA or GWAS have provided important insights into genetic processes underlying climate adaptation. For example, the *Drosophila* and *Arabidopsis* models have a long tradition in the study of climate adaptation, with extensive knowledge of phenotypic traits and many well‐studied candidate polymorphisms along clines on different continents (Atwell et al. 2011; Fournier‐Level et al. 2011; Fabian et al. 2012; reviewed in Adrion et al. 2015). These studies have provided evidence for rapid and stable adaptive oscillations of allele frequencies over seasonal time scales (Bergland et al. 2014), the importance of inversion polymorphisms (Kapun et al. 2014, 2016), polygenic adaptation (Exposito‐Alonso et al. 2018b), migrants with beneficial alleles but also novel mutations and adaptive introgression (Hancock et al. 2011; Flood and Hancock 2017) in climate adaptation.

Sequencing entire genomes is important, since climate adaptation might not be restricted to single genomic loci but might depend on larger genomic regions including structural variants such as inversions. With decreasing sequencing costs and improved genome assembly and analysis algorithms, it is now feasible to perform WGS studies also in nonmodel species such as lichens (lichen‐forming fungus *Lasallia pustulata*, Dal Grande et al. 2017), insects *(Anopheles gambiae*, Cheng et al. 2012; *Apis mellifera*, Chen et al. 2016; *Chironomus riparius*, Waldvogel et al. 2018), molluscs (*Crassostrea gigas*, Zhang et al. 2012), and vertebrates (domesticated sheep, Yang et al. 2016).

#### Time‐for‐time approach

While the space‐for‐time approach will only deliver approximations and might be confounded by covariation, the time‐for‐ time approach offers the possibility to track evolutionary changes directly. To this end, *evolve and re‐sequence (E&R) studies* typically combine WGS of individuals or populations with experimental evolution (Kofler and Schlötterer 2013; Long et al. 2015). We can experimentally follow evolutionary trajectories of individuals during an environmental perturbation. Temporal genome analyses allow us to disentangle even weakly selected loci from genomic background noise. Given that the organism is experimentally amenable (e.g., sufficient number of individuals, appropriate number of generations to allow for sufficient recombination), E&R can deliver the highest level of genomic resolution of all currently used approaches. With such data, we can test whether populations carry sufficient standing genetic variation to adapt to climate change, or we can identify the genomic architecture of climate adaptation (e.g., polygenic patterns vs. selective sweeps at a few loci with large effect).

Again, the *Drosophila* model has pioneered E&R studies, providing important insights into the evolution of climate adaptation: evolution of *Drosophila melanogaster* populations under hot climate conditions resulted in genetic adaptation and was not due to phenotypic plasticity (Orozco‐Terwengel et al. 2012; Tobler et al. 2014); adaptation seemed to be polygenic and with different genomic footprints underlying similar adaptive phenotypic changes among replicates (Barghi et al. 2019). With genome‐wide scans in evolving *Drosophila simulans* populations, it was moreover possible to identify a central metabolic switch as key factor for thermal adaptation (Mallard et al. 2018). Large‐scale and long‐term experimental evolution to search for genomic innovation is broadly applicable in bacteria and an E&R study with *Escherichia coli* populations revealed detailed insights in adaptation to temperature: during the evolution to different temperature regimes, genomic signatures of adaptation were found to be highly specific with 17% overlap in mutated genes in strains that evolved under the same regime (Deatherage et al. 2017). Parallel evolutionary responses to the same environments are often observed at the level of genes, operons, and functional complexes, but less so at the nucleotide level (Tenaillon et al. 2012, 2016). Modes of adaptation of polygenic traits can either lead to convergence or genomic redundancy among individual genomes. In the latter case, it will be difficult to derive general pathways to adaptation. Exploring the likelihood for convergence or redundancy will, thus, pose a challenge to population genomicists and bear high potential for analytical innovation in the field.

E&R experiments have certain limitations. They remain artificial and cannot reflect ecological reality, for instance, they neglect complex selection regimes, community effects, and different migration scenarios. Furthermore, they require an extensive experimental set‐up (Kofler and Schlötterer 2013; Schlötterer et al. 2015), and come with many technical complications (e.g., large variation between replicates, increased mutation rates, and unconscious selection bias). Thus, E&R studies are hardly feasible for most multicellular organisms with long generation times. Furthermore, many organisms are partially asexual or selfing, and will, therefore, show limited amounts of recombination over the course of an E&R experiment.

Instead of performing complex E&R experiments under laboratory conditions with all their limitations, there is also naturally occurring data that allow the study of evolutionary change at the genome level over evolutionary timescales. This includes remains of zooplankton in dateable sediments (e.g., Brede et al. 2009; Rellstab et al. 2011; Orsini et al. 2012), water flea individuals resurrected from resting stages in biological archives (e.g., Decaestecker et al. 2007), historical samples from archives (e.g., archived otholits Therkildsen et al. 2013b), herbarium specimens (Lang et al. 2019), or trees of different ages inferred by dendrochronological methods (e.g., Jump et al. 2006; Elleouet and Aitken 2018). Especially, resurrection experiments offer the possibility to span longer evolutionary timescales when dormant propagules (e.g., ephippia, seeds) from past times are retrieved from nature and compared to descendant individuals from the same localities in a common garden (Franks et al. 2018). These approaches have been mainly used in the pregenomic era (see Therkildsen et al. 2019), but could be easily analysed with WGS (Bálint et al. 2018). Herbarium specimens of *Arabidopsis thaliana*, for instance, have been used to study the emergence of de novo mutations through time (Exposito‐Alonso et al. 2018a).

However, time‐for‐time analyses, either based on E&R experiments or on time‐series data collected from natural populations, can only be applied to a small proportion of species and are, thus, less relevant for the broad‐scale assessment of biodiversity responses to climate change.

### EXTINCTION RISK DUE TO DEMOGRAPHIC DECLINE

The potential of a species to genetically adapt to changed environmental conditions depends on the level of genetic diversity of a population in a respective habitat, the extent of migration and gene flow between populations, and the rate of genetic innovation (e.g., by de novo mutations). If incoming gene flow and dispersal are limited, rapid environmental changes can lead to dramatic demographic shifts (especially in small populations) and only genetic adaptation can restore positive population growth and allow for the so‐called evolutionary rescue of the population (Carlson et al. 2014). Adaptation from standing genetic variation is particularly important under rapid environmental change (as due to GCC) because evolution can act on alleles that already segregate at higher frequencies in the population (speed of selection depending on the effective population size) and which can carry an adaptive value under new environmental conditions (Tigano and Friesen 2016).

Genomic data can provide information on within‐species genetic variation, a species’ demography, and effective population size (*N_e_*). These population genomic parameters can be estimated with high confidence and serve as proxies or estimators of the vulnerability of a population or species to succumb to changing climate conditions (Fitzpatrick and Keller 2015; Rellstab et al. 2016; Bay et al. 2018; Exposito‐Alonso et al. 2018b)—a major step toward predicting evolutionary responses.

## Prediction Models of Species Responses to Global Climate Change

### PREDICTING RANGE SHIFTS WITH AND WITHOUT EVOLUTION

Most studies forecasting biodiversity responses to climate change currently use statistical and functional *species distribution models* (hereafter *SDMs*; broadly including niche, envelope, and bioclimatic models). In brief, they correlate geographic location data of a species’ occurrences and experimentally gained functional models with a comprehensive set of climatic variables (Pearman et al. 2008; Elith and Leathwick 2009). This ecological modelling approach yields a coarse estimation of current or future Grinellian niche dimensions of a species, depending on the statistical association of species occurrences and environmental data. However, these models generally consider species as uniform and static with regards to climate‐relevant traits, and disregard intraspecific variation, local adaptation, and genetic potential for rapid evolutionary change (Jay et al. 2012).

Evolutionary models rely on quantitative genetics theory to estimate trait heritability. Such models have been used in animal and plant breeding, as well as in predicting responses to selection of nondomestic species including responses to changing climate conditions (e.g., Alberto et al. 2013; Gonzalez et al. 2013). It was possible, for instance, to reveal a lack of adaptive potential in a threatened New Zealand bird species by combining quantitative genetics with long‐term phenotypic data and fitness proxies (de Villemereuil et al. 2019). It has further been shown that SDMs lacking quantitative genetics can vastly underestimate species range dynamics, for example, range expansions in *Aedes aegypti*, the mosquito that transmits dengue virus (Kearney et al. 2009).

To improve predictions of climate responses, quantitative genetics should be an integral part of modelling frameworks. However, obtaining pedigrees and documentation of phenotypic key traits is time consuming and only possible in a handful of species. These species must be amenable to controlled crosses, common garden experiments, and/or long‐term individual monitoring (e.g., birds, large mammals). Moreover, fitness traits have to be identified, and ideally experimentally confirmed (Shaw 2019).

The integration of ecological and evolutionary models can potentially lead to more realistic predictions of species' persistence under GCC (Hoffmann and Sgrò 2011; Urban et al. 2016; Benito Garzón et al. 2019). Today, several models incorporate genetic or evolutionary information within SDMs (e.g., hybrid SDMs; Dormann et al. 2012). For example, genomic hybrid SDMs use geographical covariation of SNP frequencies with environmental variation, basically subdividing the species’ distribution into genetic clusters associated with climatic conditions (Fitzpatrick and Keller 2015; Exposito‐Alonso et al. 2018b; Razgour et al. 2018, 2019; Lowry et al. 2019). By building one SDM per SNP and using joint projections of the respective SNP niches into future climates (Exposito‐Alonso et al. 2018b) or by calculating genetic distances based on many SNPs along environmental gradients (Fitzpatrick and Keller 2015; Rellstab et al. 2016; Bay et al. 2018), these approaches attempt to assess mismatches between current allelic compositions and predicted future local conditions, assuming that local populations are currently adapted to their environment. Such gene‐environment mismatches are sometimes called genomic vulnerability, genetic offset, or risk of maladaptation (see above and Rellstab et al. 2016; Bay et al. 2018).

However, hybrid SDMs remain a static association of genetic clusters with environmental variation. Genomic vulnerability as such, only refers to the mismatch of current allele frequencies with potential future climatic conditions. It does not incorporate predictions of shifts in allele frequencies caused by selection or gene flow among populations. When explicitly taken into account, migration and selection may decrease the mismatch of current genomic compositions and future abiotic conditions, and lead to a prediction of lower genomic vulnerability (Exposito‐Alonso et al. 2018b). Furthermore, measures of genomic vulnerability cannot directly predict whether a species will adapt or succumb to extinction, as it does not include information on evolutionary rates, adaptive phenotypic changes, or future population growth rates (i.e., absolute fitness). This is caused by the general lack of estimates of effect size of the genetic variants associated with environmental variables (but see Taylor et al. 2019). Hybrid SDMs are therefore unable to model local population density as a function of the degree of local adaptation. Nevertheless, genomic data can provide the demographic information needed to make more accurate predictions. In particular, spatial patterns of genetic diversity can provide estimates of migration rates and effective population sizes required to parametrize process‐based models (see below). Moreover, when coupled with past climatic information, models of temporal change in population density can be constructed with coalescent‐based simulations. By associating past changes in population density with past changes in climatic conditions, demographic models can then be validated by hindcasting (see Brown et al. 2016; Prates et al. 2016).

### PREDICTING ECO‐EVOLUTIONARY RESPONSES OF BIODIVERSITY TO CLIMATE CHANGE

Current hybrid SDMs do not fully integrate eco‐evolutionary dynamics, but rather a subset of the processes involved. For instance, some approaches incorporate migration and demography, but not evolution (e.g., Dullinger et al. 2012), or dispersal and evolutionary trait dynamics, but not demography (Bush et al. 2016), or climate‐driven selection of genetic variants, but not demography and migration (Exposito‐Alonso et al. 2019). To predict species’ range shifts, other approaches couple mechanistic, process‐based, physiological, or phenological models with information on evolutionary adaptation (Kearney et al. 2009; Oddou‐Muratorio and Davi 2014) or phenological plasticity (Wilczek et al. 2010; Duputié et al. 2015). Only recently, Cotto et al. (2017) have developed a full, individual‐based *eco‐evolutionary model (EEM)* of adaptation to climatic changes that integrates quantitative genetics, thus, disregarding specific loci. EEMs are process‐based in which they take into account life‐history traits and genetic characteristics of a species, and simulate population dynamics and evolution on a spatial grid for a given scenario of environmental change. This approach requires extensive data on the evolutionary potential of a species (genetic covariance of phenotypic traits, strength of selection), its spatial occurrence and ecological characteristics (e.g., dispersal kernels, vital rates). EEMs are, thus, bound to be limited to a handful of species, but can deliver valuable information on species’ extinction risks in specific geographical areas. Given appropriate computational resources, and adequate approximations, individual‐based (e.g., Guillaume and Rougemont 2006) or population‐based (e.g., Dullinger et al. 2012; Bush et al. 2016) simulation approaches offer an exciting avenue to build EEMs for forecasting species’ responses to climate change. Nevertheless, the genetic information these models require is in the form of additive genetic variance or heritability of quantitative traits, or, even better, genetic covariation of traits with fitness. Thus, there is a disconnect with the previous genetic/genomic hybrid SDM approach because information on allele frequencies across environmental space is directly transformed to changes across time without translating it into adaptive genetic variation of traits, and temporal demographic feedbacks, as EEMs do. One avenue to connect these two approaches is to utilize genomic SDMs to define parameters across space needed for EEMs.

The predictive potential of EEMs can be leveraged by a better integration with ecological genomics. The discipline of ecological genomics combines classical ecological research with population genomic approaches in order to study the genetic basis underlying responses of organisms to variation in their natural environment (Ungerer et al. 2008). Genomic data can provide EEMs with estimates of quantitative genetics parameters from natural populations sampled across environmental gradients (Gienapp et al. 2017). Traditionally, relatedness between individuals has been estimated from pedigrees or from controlled crosses in common gardens. Although not yet widely applied, so called “*genomic quantitative genetics*” (*gQG*) relies on estimates of genetic relatedness between individuals that are based on genome‐wide polymorphisms (see Speed and Balding 2015 for an overview; see Gienapp et al. 2019 for an example in birds). Phenotypic trait covariation between individuals with known levels of relatedness is then used to estimate the additive genetic covariation of a set of quantitative traits. The response of a population to a shift in local trait optima (e.g., caused by climate change) can then be predicted from the additive genetic covariance between traits and fitness, given that the strength of selection is known (Etterson and Shaw 2001). Even though gQG can help extending the quantitative genetics approach of EEMs to any wild species, the need for phenotypic data and reliable fitness estimates across environments remains. For some species where cohort or time‐series data are available, knowledge of the genetic relatedness among individuals through time can help identify the number of surviving offspring per reproducing adult (Truffaut et al. 2017). Estimates of reproductive success can then be related to phenotypes in order to estimate the strength of selection within populations; or it can directly deliver an estimate of the species’ evolvability from the genetic additive variation in fitness. This will still require large sampling efforts. Further developments of the gQG approach are, thus, necessary to identify the best sampling strategies to allow for its application in wild species. Nevertheless, gQG has the potential to yield estimates of the evolutionary potential of wild species for which classical breeding plans or pedigree information are not accessible. When quantitative genetic parameters are not available, the EEM approach has the advantage to allow for sensitivity analyses on eco‐evolutionary parameter values and deliver process‐based estimates of extinction probabilities. Doing so will allow deploying EEM approaches to a broader range of species in natural contexts.

## Challenges and Outlook

The severe impact of GCC on biodiversity necessitates the development of predictive models that can help to take timely actions (Urban 2015). Current approaches are limited by either technical issues (e.g., statistical methods that integrate genomic and environmental information) or data acquisition challenges (e.g., data availability). Here, we have highlighted some recent technical advances that integrate genetic information for ecological projections, and identified major challenges in the area of data acquisition for a wide range of organisms (see above and Fitzpatrick and Keller 2015; Bush et al. 2016; Rellstab et al. 2016; Cotto et al. 2017; Exposito‐Alonso et al. 2018b).

**Figure 2:**
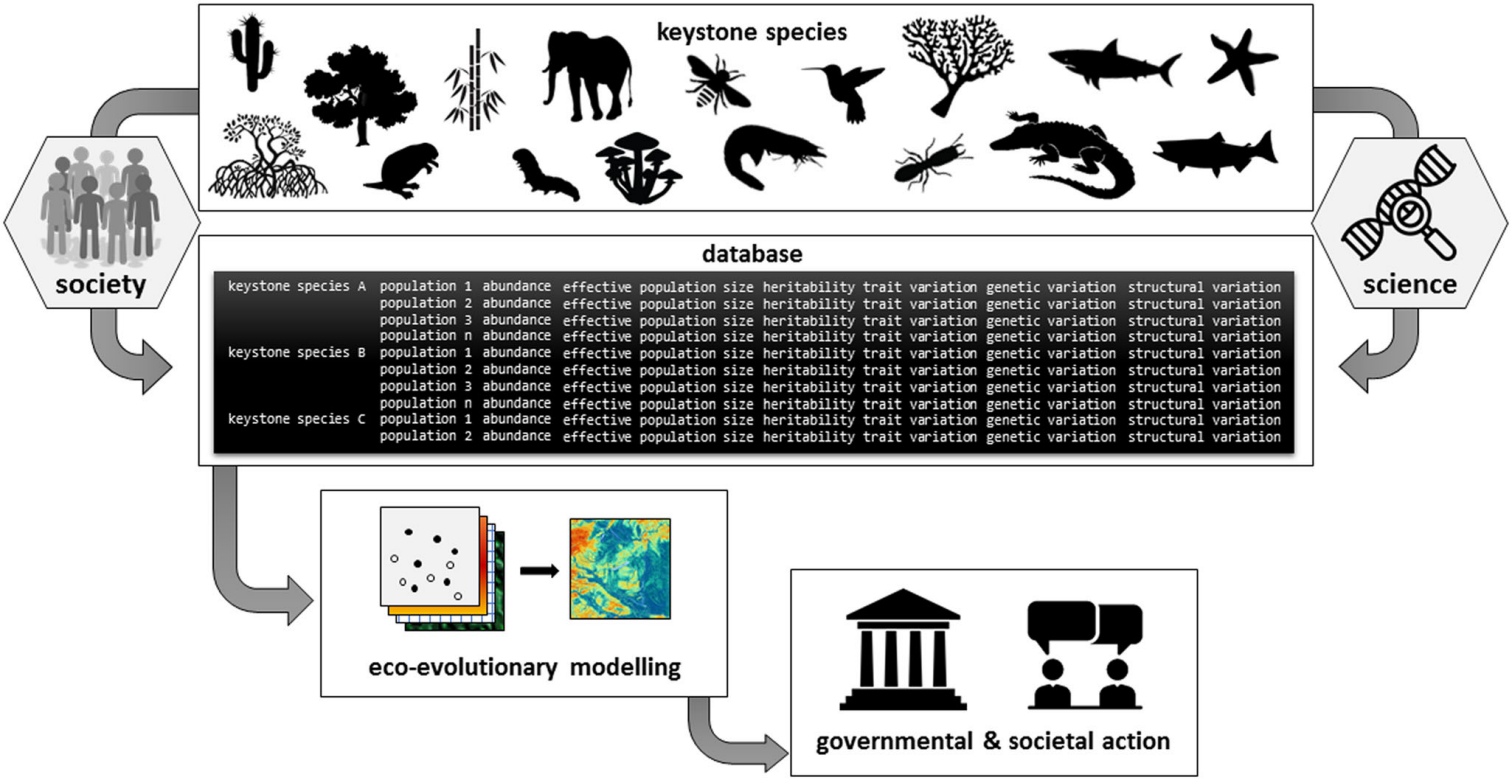
Outline of our proposed mode of action to collect, store, and analyse eco‐evolutionary data as a joint venture of **citizens** and **scientists**. A comprehensive **database** can then support **eco‐evolutionary modelling** to predict biodiversity responses to GCC. These predictions will provide **government** and **society** with educated recommendations in order to take significant actions for conservation.

Realistic eco‐evolutionary prediction models of species distributions require large amounts of data, magnitudes higher than what is currently available. Thus, several logistical challenges have to be addressed to build and expand databases for environmental data, species abundance data, and genomic data on a global scale (Fig. 2). Environmental data, and especially climate data, are already available in public databases with sufficient resolution for large parts of the globe (e.g., WorldClim2, Fick and Hijmans 2017; or CHELSA, Karger et al. 2017). Species’ distribution records are publicly available for an increasing number of species (GBIF.org). Future research could also draw from satellite and remote sensing data to build an analytical platform for near‐term prediction of habitat change. Automatic platforms that use SDMs could combine the abundance data with environmental and land use systems (worldclim.org, CORINE, or USGS Land Cover) to produce current geographic distributions (GFBIO.org, IUCN) and predict changes in geographic distribution boundaries.

Combined with corresponding phenotypic variation or geographical and ecological information, genomic data can help estimating some of the evolutionary quantities relevant for eco‐evolutionary forecasting. Genomic data is constantly accumulating in public databases, such as NCBI and EMBL‐EBI, however there is a strong imbalance of sequencing model versus nonmodel species and resequencing of laboratory versus natural populations. Databases will grow even faster as soon as portable devices allow ecologists to sequence directly in the field (Michael et al. 2018), as is routinely done for trait, photographic, or environmental data. Even though genome sequencing is improving in terms of cost and portability (https://www.genome.gov/sequencingcostsdata/), so far sequencing effort is still focusing on an unrepresentative fraction of biodiversity, that is, mainly few (model) species. In the future, however, genetic monitoring for conservation requires a systematic approach that encompasses relevant biodiversity (Reside et al. 2018). To address this challenge, sequencing effort may prioritise keystone species, which might inform about ecosystem‐wide evolutionary responses (Fig. 1). Keystone species act as major ecosystem hubs, for example, the most predominant tree species in a forest, and their decline or major range shifts would most likely generate cascading effects that affect the whole ecosystem (Mills et al. 1993; Valls et al. 2015). Studying networks of species interactions can help to identify keystone species (Bascompte et al. 2006; Tylianakis et al. 2008). Another set of high‐priority species should be those flagged as being threatened in the red list of the International Union for Conservation of Nature (https://www.iucnredlist.org/), as arguably those are undergoing early impacts.

The overall mission will be to comprehensively collect occurrence and abundance data of the prioritised set of species and finally sample populations across the species’ distribution range. In order to realize this task on a short‐term time scale, the involvement of citizens bears high potential. From plant ecology experiments (NutNet, DryNet), to the worldwide “watch” on any species (iNaturalist or iSpot), citizen science‐based platforms open the door for global‐scale scientific databases. Citizens can even be involved in sampling efforts, when integrated in well‐organised scientific projects that deal with distribution of required sampling material and the compliance with international legislation concerning biological samples. On different scales, such citizen science projects have already proven to be highly successful, as for example, the EcoAction program to survey the health of coral reefs around the world (reefcheck.org, Done et al. 2017) or the “big wasp survey” to sample wasp populations across the United Kingdom (bigwaspsurvey.org, Sumner et al. 2019).

Genomic sequencing of the collected samples can be realised by scientific consortia that integrate genome sequencing centres, as there already exists, for example, the Earth Biogenome Project (earthbiogenome.org, Lewin et al. 2018) and the Darwin Tree of Life UK project (sanger.ac.uk/news/view/genetic‐code‐66000‐uk‐species‐be‐sequenced). The ultimate aim would be to establish platforms that integrate different databases (climate data, species abundance data, phenotypic data, genomic data, etc.; Fig. 2) with the necessary analysis pipelines, to produce publicly available and easily interpretable species vulnerability projections. A successful example that has pioneered large‐scale community‐based data collection comes from human‐pathogenic organisms as Ebola, Flu, or Zika, where researchers have shared significant amounts of data during critical outbreaks and analyses were generated and published in almost real time (http://nextstrain.org). New online technologies facilitate forming consortia of citizens and scientists that can coordinate, process, and share data in safe and reproducible ways. We think that these new ways of collecting and processing data are promising avenues for improving our understanding of the limits of species tolerances and adaptation and might, thus, help to parametrize the next generation of species response models (Swan et al. 2010; Özdemir et al. 2013; Grossman 2019).

As soon as projections on the responses of biodiversity to climate change have reached nationwide, continental or even global scales, these results gain in importance to become integrated in political decision making. Publicly available databases will sensitise citizens for the effects of GCC on biodiversity and, thus, strengthen the need for political awareness, especially on local scale. Results can furthermore be integrated in reports of intergovernmental panels like IPCC and/or IPBES (ipcc.ch, ipbes.net) in order to use already established and acknowledged channels to policymakers.

## Conclusion

GCC poses severe risks to biodiversity and latest extrapolations alert an extinction risk due to climate change for more than 5% of all species from 2°C global warming alone (IPBES 2019). We, thus, urgently need to better understand and accurately predict how species respond to changing environmental conditions in order to inform policymakers and implement conservation strategies. We need to incorporate ecological as well as evolutionary parameters in our models to account for a species’ potential to compensate changing environmental conditions by either range shift or genetic evolution for adaptation. A species’ evolutionary potential for adaptation can be estimated from genomic data. Hybrid SDMs, which attempt to at least partially incorporate eco‐evolutionary dynamics, are becoming more frequent and deliver informed estimates of a species' vulnerability. EEM that incorporate gQG approaches can yield more realistic predictions and be applied to any wild species. Such models require extensive amounts of data and especially the required genomic data could be generated rapidly enough (i.e., without too much experimental effort) and for a representative large group of taxa (e.g., keystone species). We, here propose a roadmap of how science and society can work together to facilitate sampling, estimating of fitness parameters, and genome sequencing for a broad range of species to meet the urgent need of action in face of the accelerating speed of GCC.

Associate Editor: Z. Gompert
